# In situ observations show vertical community structure of pelagic fauna in the eastern tropical North Atlantic off Cape Verde

**DOI:** 10.1038/s41598-020-78255-9

**Published:** 2020-12-11

**Authors:** H. J. T. Hoving, P. Neitzel, H. Hauss, S. Christiansen, R. Kiko, B. H. Robison, P. Silva, A. Körtzinger

**Affiliations:** 1 GEOMAR Helmholtz Centre for Ocean Research Kiel, Düsternbrooker Weg 20, 24105 Kiel, Germany; 2 Christian Albrecht University Kiel, Christian-Albrechts-Platz 4, 24118 Kiel, Germany; 3 University of Oslo, Blindernveien 31, 0371 Oslo, Norway; 4 Laboratoire dOcéanographie de Villefranche, Sorbonne Université, Villefranche-sur-Mer, France; 5 Monterey Bay Aquarium Research Institute, Sandholtroad 7700, Moss Landing, USA; 6 Ocean Science Centre Mindelo & Instituto do Mar (IMAR), Cova de Inglesa, C.P. 132 Mindelo, São Vicente Republic of Cabo Verde

**Keywords:** Ecology, Zoology, Ocean sciences

## Abstract

Distribution patterns of fragile gelatinous fauna in the open ocean remain scarcely documented. Using epi-and mesopelagic video transects in the eastern tropical North Atlantic, which features a mild but intensifying midwater oxygen minimum zone (OMZ), we established one of the first regional observations of diversity and abundance of large gelatinous zooplankton. We quantified the day and night vertical distribution of 46 taxa in relation to environmental conditions. While distribution may be driven by multiple factors, abundance peaks of individual taxa were observed in the OMZ core, both above and below the OMZ, only above, or only below the OMZ whereas some taxa did not have an obvious distribution pattern. In the eastern eropical North Atlantic, OMZ expansion in the course of global climate change may detrimentally impact taxa that avoid low oxygen concentrations (*Beroe*, doliolids), but favour taxa that occur in the OMZ (*Lilyopsis*, phaeodarians, Cydippida, *Colobonema*, *Haliscera conica* and *Halitrephes)* as their habitat volume might increase. While future efforts need to focus on physiology and taxonomy of pelagic fauna in the study region, our study presents biodiversity and distribution data for the regional epi- and mesopelagic zones of Cape Verde providing a regional baseline to monitor how climate change may impact the largest habitat on the planet, the deep pelagic realm.

## Introduction

The meso-, bathy- and abyssopelagic ocean—the water column below the sunlit euphotic zone—is the largest biome for multicellular organisms on the planet, and is the habitat with the highest faunal biomass as well as the greatest number of individual organisms^1,2^. Biological observations and measurements in the deep water column are lacking for many geographical regions, but they are required for an understanding of functional biodiversity and for securing sustainable provision of deep-sea ecosystem services^3^. The oceans are changing at an unprecedented pace with marine ecosystems being impacted by major stressors including global warming, pollution, acidification, overfishing and the decline of dissolved oxygen concentrations in the water column (deoxygenation)^4–7^. The absence of regional biological baselines in the pelagic ocean hampers the evaluation of the impact of these stressors^8^.

In regions with high surface productivity (and, thus, high export flux) and restricted ventilation (in particular in the globe’s four eastern boundary current systems), dissolved oxygen profiles follow a distinct pattern, with a minimum at mesopelagic depths^9^ that form oxygen minimum zones (OMZs). The passive sinking of marine snow to mesopelagic depths results in high abundance of organic matter, which is consumed and respired by both metazoans and microbes involving the consumption of oxygen^10^. Mesopelagic oxygen minimum zones are present in all oceans, but their vertical extent, core depth and minimum oxygen concentration differ between regions^11,12^. The most pronounced mesopelagic OMZs occur in the eastern tropical Pacific and in the Arabian Sea, with oxygen concentrations dropping below the detection limit in some cases^11^. In the eastern tropical North Atlantic (ETNA), the OMZ is centered between approximately 300 m and 600 m depth, with a minimum oxygen concentration around 35–40 µmol kg^−1^ in the core^13^. The global ocean is losing oxygen: the oxygen concentrations in OMZ cores are declining, the vertical extents of OMZs are expanding and their horizontal reach is increasing. The result is an overall increase of OMZ volume with more intense cores^6,7^. Oxygen loss in the water column may be increased in areas with ocean warming, narrowing that part of the habitat with viable conditions for many species^14^.

Oxygen is vital for metazoan life and hence is a major driver of biogeographical patterns^15^. Many organisms have evolved remarkable adaptations to efficiently extract oxygen from the water column, allowing them to permanently or temporarily endure OMZ conditions^16^. The first trait is to efficiently extract oxygen from the water, the second is the ability to temporarily reduce metabolic rates when confronted with low oxygen partial pressures, and the third is to use anaerobic metabolism when necessary. To facilitate these traits, organisms have evolved specific morphologies and behavioral patterns. Fishes, cephalopods and crustaceans inhabiting the OMZ may have increased gill surface area to facilitate oxygen uptake or have a relatively low metabolic rate^17^. Additionally, these organisms may visit the OMZ only temporarily, e.g. for hunting or refuge, and then migrate back again to more oxygenated waters to compensate their anaerobic metabolic debt^18^. Temporary visits may be part of the daily vertical migration. Many zooplankton and nektonic organisms hide within the mesopelagic “twilight” zone during the daylight hours to avoid visual predators and ascend at night to the productive epipelagic zone to feed^19^. This diel vertical migration (DVM) of zooplankton and nekton contributes to a net downward flux of particulate and dissolved matter^20,21^. However, the mesopelagic zone also contains a specific set of resident organisms that do not migrate to the surface, and instead feed on sinking particles and the migrant organisms^22,23^. Besides the comparatively well-known migration behavior of crustaceans and fishes, some gelatinous organisms such as pyrosomes and salps also migrate on a daily basis^24^, but more observations are needed to quantify taxon-specific distribution and migration patterns.

Most animals are physiologically stressed when exposed to reduced oxygen levels and hence OMZs create ecological zones in the water column. For example, the mesozooplankton distributions in the eastern tropical Pacific show well defined accumulations of biomass at the thermocline and the lower oxycline^25^. In Monterey Bay, diversity and abundance of nekton and macrozooplankton are generally reduced inside the core of the OMZ but some ctenophores, bathylagid fishes, and cephalopods cope well with the OMZ^26,27^. Squids in the Pacific (*Gonatus* spp., *Dosidicus gigas*) and the Atlantic (*Sthenoteuthis pteropus*) temporarily visit the OMZ to hunt for myctophid fishes, which find refuge in the upper OMZ during the day^26,28,29^. Cranchiid squids in Monterey Bay show a distinct bimodal vertical distribution bracketing the OMZ^27^. Atlantic billfishes forage for squids and myctophids, principally in the layers above the OMZ, since their relatively high metabolic demands do not allow venturing into OMZs^30^. As a result, the expansion of the Atlantic OMZs has resulted in habitat reduction for billfish in the eastern tropical Atlantic^31^.

Faunal responses to OMZ expansion are taxon-specific and depend on thresholds of hypoxia tolerance. Gelatinous zooplankton are ecologically important, delicate and abundant animals. Although data on physiology and metabolic rates in gelatinous fauna are limited, some have low metabolic rates and the potential for oxygen storage in their mesoglea^32,33^. These traits may enable them to outcompete other plankton and fish in regions with eutrophication-induced hypoxia^34^, and hence potentially allow them to better cope with OMZ expansion^15^. In the ETNA, gelatinous organisms (including polychaetes) have been shown to withstand the extreme conditions in low-oxygen mesoscale eddies^22,35^. Due to their fragility, gelatinous organisms are problematic to capture and/or to identify in fixed samples from nets or pumps, and are typically underrepresented in biological surveys. Alternative means are necessary to quantitatively assess them and the enormous midwater biomass and diversity they represent.

In situ observations using submersibles, where animals are recorded alive in their natural habitat, have resulted in the discovery of a great diversity of large (> 1 cm) gelatinous zooplankton in the deep sea (e.g. *Deepstaria enigmata*^36^; *Kiyohimea usagi*^37^; *Tiburonia granrojo*^38^). They also have revealed new information on diversity^39^, organism associations^40^; vertical zonation^41^, seasonality^42^, trophic interactions^43^, and have facilitated major taxonomic revisions^44^ . Quantitative vertical distribution information on gelatinous fauna can be obtained via horizontal video transect methodology, where faunal observations and measurements are linked with hydrographic data. Using ROVs, such efforts have led to community assessments of gelatinous fauna under Arctic ice^45^, on the mid-Atlantic Ridge^46^, in the Indian Ocean^47^ , off Japan^39^, and in the meso- and bathypelagic zones of Monterey Bay^41,48^. To evaluate the potential consequences of ocean change in the deep sea, gelatinous fauna must be considered, as they are pivotal and abundant components of oceanic food webs^1,43,49^. While observational data of vertical distribution patterns alone cannot provide a causal link to driving parameters, they provide insight into the habitat zonation of the fauna and allow discussion regarding distribution in relation to habitat features, e.g. tolerance of low oxygen encountered in the OMZ.

Using a towed deep-sea pelagic observation system^50^ and by applying video transect methodology in the water column down to 1000 m of depth, we present baseline observations of gelatinous organism distribution in the ETNA in relation to the oxygen distribution, as well as other environmental drivers (temperature, chlorophyll-a, predator and prey abundance). We use these data to test the hypothesis that gelatinous fauna inhabit the ETNA OMZ. Additionally, we present detailed distribution patterns including daily vertical migration behaviour and discuss potential taxon-specific consequences of OMZ expansion. Although the taxonomic resolution of identifications from the video transects were limited, the presented in situ data are among the first for large gelatinous zooplankton biodiversity in the Cape Verde region and will help to elucidate the role of gelatinous animals in the biological carbon pump.

## Results

### Regional oceanography

**Figure 1:**
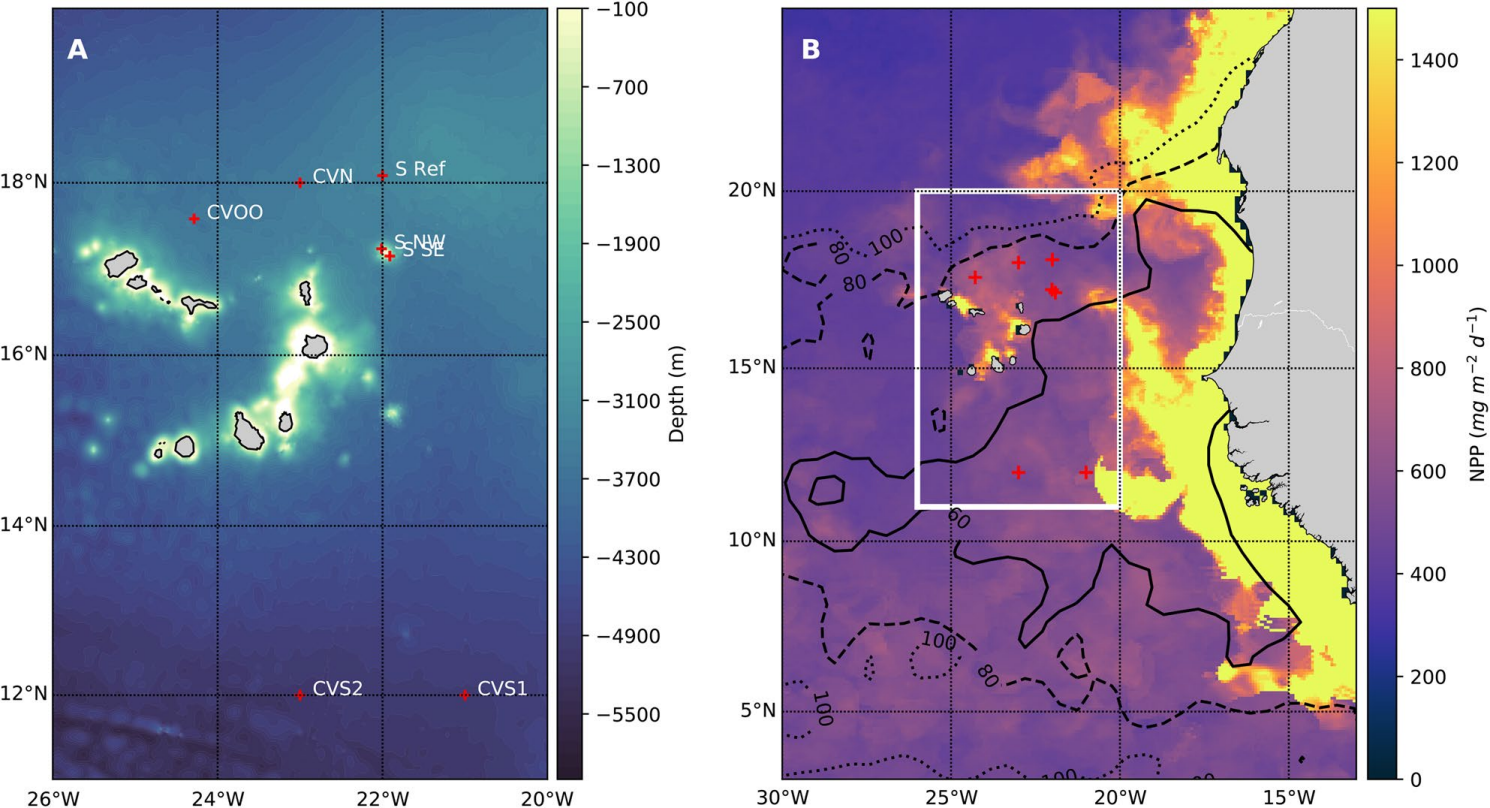
The eastern tropical North Atlantic (**A**). Zoomed-in working area, as in the white box from (**B**), around Cape Verde with coded stations indicated (color scale indicates GEBCO bathymetry). CVOO Cape Verde Ocean Observatory, CVN Cape Verde North, S Ref Senghor Reference, S NW Senghor North West, SSE Senghor South East, CVS1 Cape Verde South 1, CVS2, Cape Verde South 2. (**B**). with color scale indicating satellite-derived net primary productivity (NPP) during cruise MSM49 (estimated according to^51^ and obtained from http://sites.science.oregonstate.edu/ocean.productivity), black contours indicating 60, 80 and 100 µmol kg^−1^ oxygen at 400 m depth (World Ocean Atlas 2018). Red crosses indicate working stations. Maps were produced using python (packages: matplotlib and basemap).

Transecting and hydrography stations were located near the Cape Verde islands, where bottom depths ranged between 1000 and 4900 m (Fig. 1A). The stations Senghor NW and Senghor SE were located on the flanks of the Senghor Seamount, northeast of Cape Verde. The time series station Cape Verde Ocean Observatory (CVOO) was, at the time of sampling, influenced by a cyclonic eddy with slightly increased surface productivity. Overall, the productivity in the region is patchy due to transient eddy activity and island-induced upwelling (Fig. 1B).

**Figure 2:**
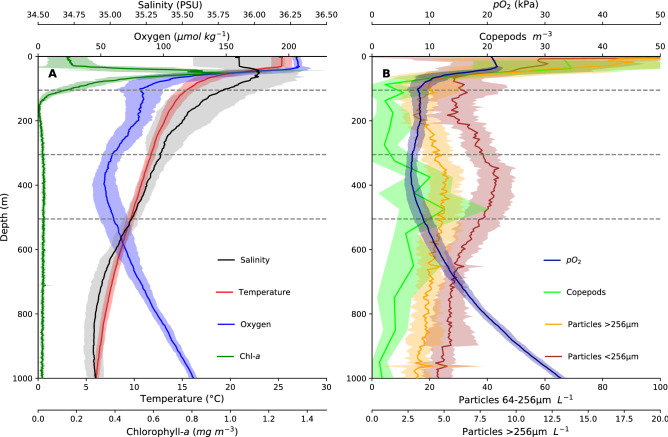
Hydrographic profiles of the sampled stations during MSM49 with mean (± SD) vertical distribution of chlorophyll-*a* (green), temperature (red) and oxygen (blue) from the CTD (left) as well as oxygen partial pressure, particle abundance (64–256 µm and > 256 µm) and copepod abundance (right). Dashed grey lines indicate four depth strata defined based upon mean hydrographic conditions (upper layer UL, upper oxycline UOC, oxygen minimum zone OMZ and lower oxycline LOC).

Mean surface temperatures in the upper layer (UL) were around 25 °C (Fig. 2). The upper limit of the pycnocline was between 30 and 60 m depth, and below that temperature steadily decreased with depth to ~ 6 °C at 1000 m. Salinity was highest between 50–70 m depth and ranged between 34.5 and 36.5.

The chlorophyll-a maximum corresponded to the pycnocline; maximum values were around 0.8–1 mg m^−3^. A moderate oxygen minimum zone (OMZ) was found at all stations at depths roughly between 300 and 500 m (Fig. 2), where oxygen concentrations were below 60 µmol kg^−1^. Minimum values of around 35 µmol kg^−1^ occurred at the southern stations. A second, shallower oxygen minimum with values around 50 µmol kg^−1^ (Fig. 2) was found at about 100 m depth at CVOO and CVSE. To analyze the vertical habitat structure, we used the following depth limits based upon the mean hydrographic conditions: upper layer (UL) 0–92 m, upper oxycline (UOC) 93–304 m, oxygen minimum zone (OMZ) 305–503 m, lower oxycline (LOC) 504–1000 m, where the upper and lower limits of the OMZ are defined by 60 µmol kg^−1^. In general, both copepod and particle abundance, indicative of food availability to particle and flux feeders as well as to predatory macrozooplankton, was highest in the UL. However, a broader, intermediate particle maximum co-occurred within the OMZ (Fig. 2).

### Fauna

**Figure 3:**
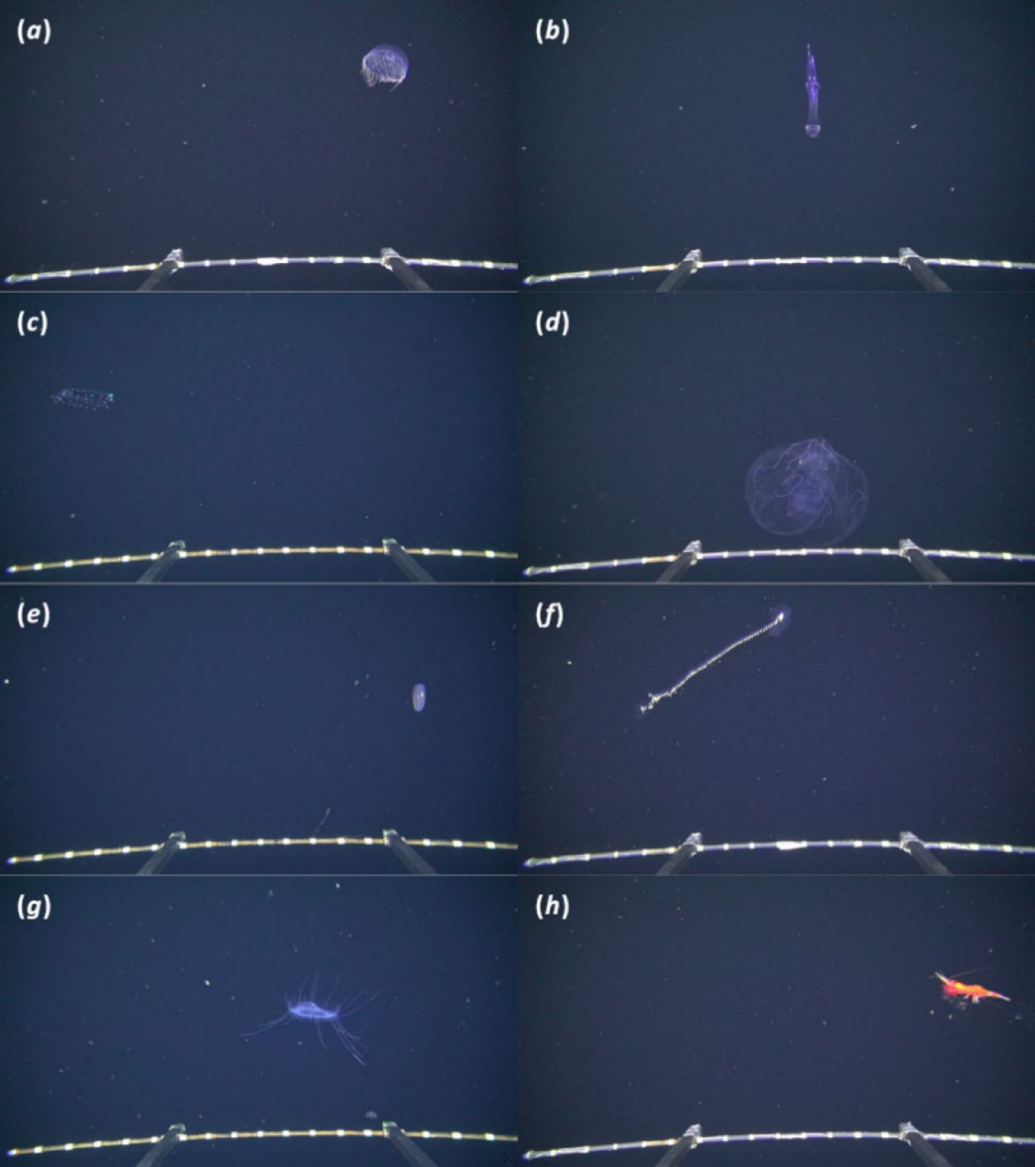
Examples of macrozooplankton as observed by PELAGIOS during MSM49 in the Cape Verde region. Organisms with peak abundances within the OMZ are (*a*) *Halitrephes* sp.^50^, (*b*) *Colobonema* sp., (*c*) *Lilyopsis* sp. and (*d*) *Thalassocalyce inconstans*. (*e*) *Beroe* sp. with a bimodal vertical distribution pattern. (*f*) *Praya*, a siphonophore with a distribution above the OMZ. (*g*) *Solmissus* sp. with a wide vertical distribution and a peak in the OMZ. (*h*) A decapod crustacean, a group of organisms that showed vertical migration. The distance between the white stripes on the bar at the bottom of the images is 5 cm.

We observed organisms from a variety of phyla including fishes, pelagic tunicates (salps, doliolids and larvaceans), crustaceans, ctenophores, medusae and siphonophores, chaetognaths, Foraminifera and Phaeodaria (Table S2; Fig. 3).

Overall, we considered 46 taxonomic groups, of which some could be identified to species level, many to genus level and some only to higher taxa (Table S2). We provide example frame-grabs from the annotated videos (https://doi.pangaea.de/10.1594/PANGAEA.919335). The five most abundant taxonomic groups were: fishes (max. 721.9 ind/1000 m^3^ at CVOO night at 50 m depth), euphausiids (max. 529.8 ind/1000 m^3^ at CVS2 day at 400 m depth), the orders of decapods and mysids combined (max. 300.4 ind/1000 m^3^ at CVS2 day at 600 m depth), and chaetognaths (563.3 ind/1000 m^3^ at CVS2 night at 100 m depth). Abundant gelatinous fauna were appendicularians (max. 965.4 ind/1000 m^3^ at CVS2 night at 50 m depth), the ctenophore *Beroe* (max. 44 ind/1000 m^3^ at SenghorSE day at 700 m depth) and the narcomedusa *Solmissus* (max. 67 ind/1000 m^3^ at CVOO day at 200 m depth). *Haliscera conica* was the most abundant trachymedusa (max. 31.2 ind/1000 m^3^ at CVS2 day at 350 m depth). Frequently observed taxa of large unicellular zooplankton were Foraminifera (max. 261.3 ind/1000 m^3^ at CVS2 night at 700 m depth) and the phaeodarians (max. 168.9 ind/1000 m^3^ at CVOO day at 400 m depth).

### Vertical distribution

**Figure 4:**
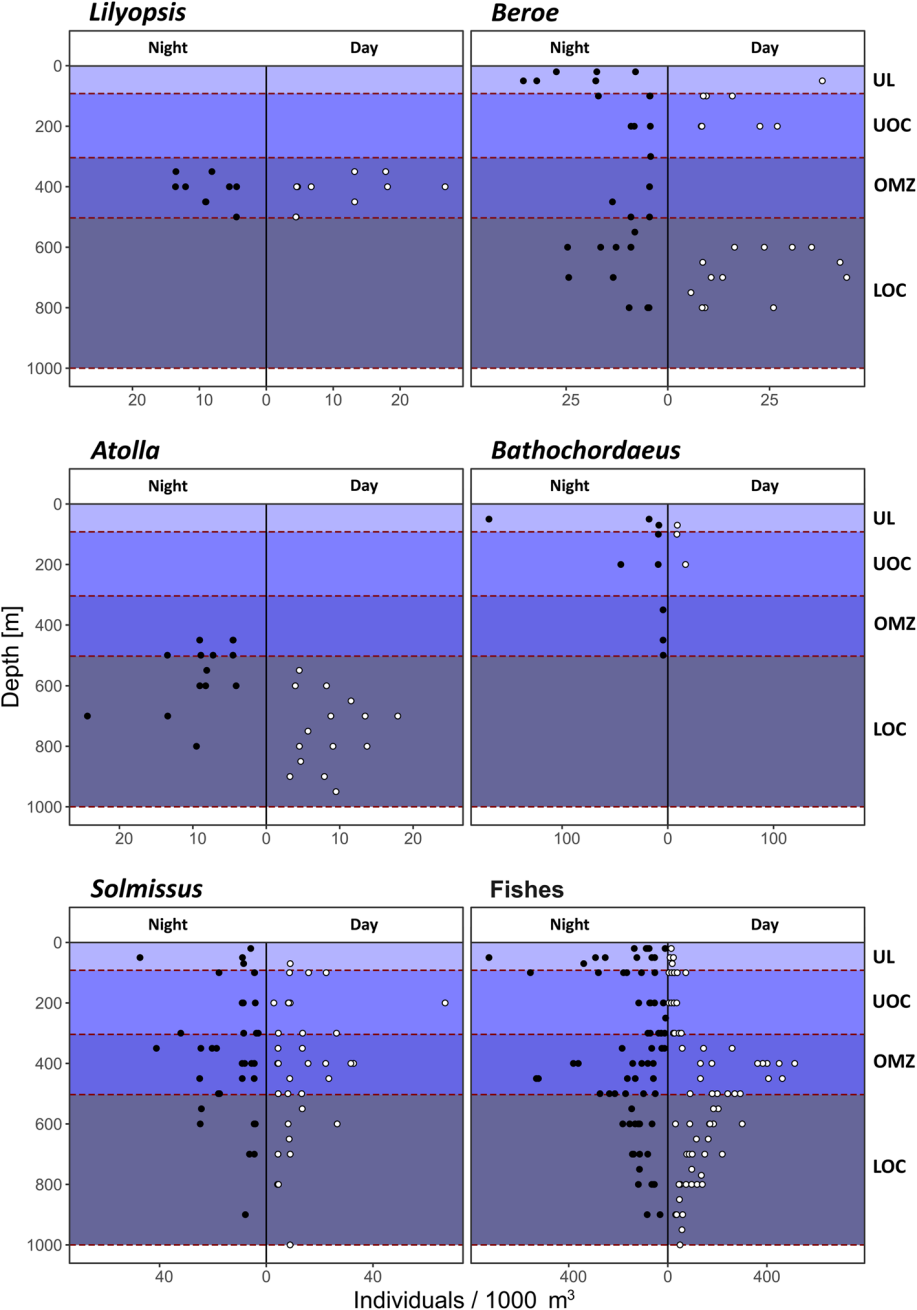
Examples of the different vertical distribution types of the faunal groups we encountered with PELAGIOS. The abbreviations refer to: upper layer (UL), upper oxycline (UOC), oxygen minimum zone (OMZ), lower oxycline (LOC), as well as sample size for night sampling (Nn) and day sampling (Dn). (*a*) *Lilyopsis* (Nn = 20; Dn = 27) with a restricted distribution that corresponds with the OMZ (Type 1), (*b*) *Beroe* (Nn = 58; Dn = 82) with a distribution that is disrupted by the OMZ (Type 2), (*c*) *Atolla* (Nn = 23; Dn = 25) with a distribution below the OMZ, including vertical migration (Type 3), (*d*) *Bathochordaeus* (Nn = 34; Dn = 4) with a distribution above the OMZ (Type 4), (*e*) *Solmissus* (Nn = 74; Dn = 88) with a wide distribution pattern that peaks in the OMZ (Type 1) and (*f*) the distribution of fishes (Nn = 1803; Dn = 1862) which shows diel vertical migration, but peaks also in the OMZ.

Taxa showed clear differences in their vertical distribution (Table S2; Figs. 4, S1). Taxa that occurred in the UL and the UOC were, for example, the giant larvacean *Bathochordaeus*, doliolids and the ctenophore *Beroe*. In the OMZ, fishes and euphausiids occurred as well as the ctenophore *Thalassocalyce inconstans*, the siphonophores *Lilyopsis*, the hydromedusae *Solmissus*, *Colobonema* and *Haliscera conica*, as well as unicellular phaeodarians. Examples of taxa that occurred below the OMZ during the day were, *Atolla*, *Crossota* and small narcomedusae potentially of the family Aeginidae.

**Figure 5:**
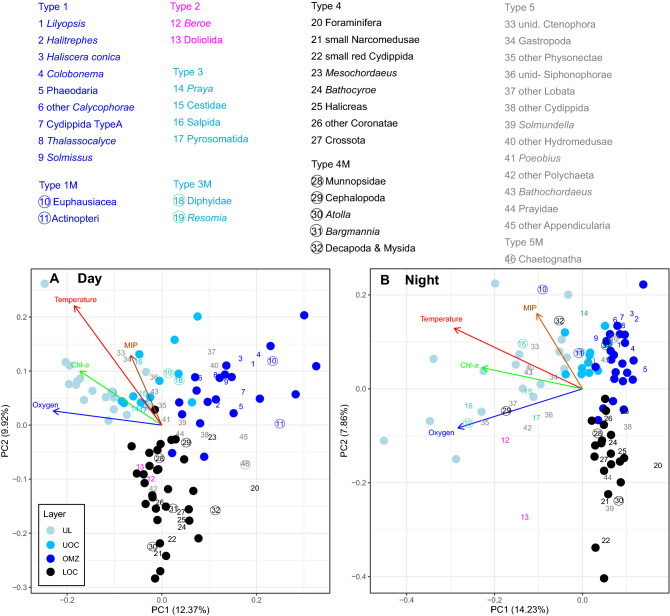
Principal component analysis of the daytime (**A**) and nighttime (**B**) community composition (46 taxa, see Table S2). Circles are samples, with the color indicating the four nominal depth layers. One sample is a discrete observation (one transect at a given station). Taxa are represented by numbers, color coded by the identified distribution type 1 (in OMZ, blue), 2 (bimodal, magenta), 3 (above OMZ, cyan), 4 (below OMZ, black) and 5 (no clear pattern, grey). Numbers in circles identify taxa with diel vertical migration (M). The mid-point of the taxon numbers is the PC1/PC2 score location. Vectors are only drawn for environmental variables (oxygen, temperature, chlorophyll-a, and particles). The layer abbreviations refer to: upper layer (UL), upper oxycline (UOC), oxygen minimum zone (OMZ), and lower oxycline (LOC).

The weighted mean depth (WMD) of occurrence was significantly different between day and night (indicative of diel vertical migration behavior) for fishes, cephalopods, decapoda and mysids, euphausiids, larvaceans, chaetognaths, *Bargmannia*, *Atolla,* dyphyid siphonophores, and the few observed munnopsid isopods (p-values in Table S2). To discern between resident and migratory species, we marked those migratory types where WMDs differed significantly between day and night with an ‘M’ (Table S2). These migratory fauna are shown by the distribution of taxa along the first two axes of the principal component analysis (Fig. 5). Decapods and mysids migrated up from daytime depths of 542–651 m to 250–316 m at night.

Euphausiids migrated from 371–499 m during the day to 138–300 m at night. Although non-significant, we detected a weighted mean depth range for *Beroe* above the OMZ from 200–83 m during daylight hours that differed from the depth of occurrence at night (100–50 m). Similarly, we observed for *Beroe* in the LOC a daytime depth range of 711–641 m which changed to 667–550 m at night. *Atolla* shifted from 652–837 m to 450–656 m. The WMD of the siphonophore *Bargmannia* shifted from 500–703 m to 100–600 m. Fishes (Actinopteri) were abundant in the upper 100 m at night but the peak of their WMD remained at around 300 m, indicating that some species or individuals did not migrate. During daytime there were nearly no fish found in the upper 100 m. The absence of a significant difference in weighted mean depth in day and night distribution of pyrosomes and salps is likely due to low sample size (both transects with occurrence and total number of specimens observed) as the PCA does suggest migration behaviour in both taxa, with the daytime depth in the UOC and the nighttime depth in the UL.

### Fauna in relation to the OMZ

The vertical distribution ranges for the taxonomic groups examined here (Table S2) can be divided into five basic types (Figs. 4, 5), which all included both particle feeders as well as predators. Type 1) the distribution peak is in the OMZ, Type 2) the distribution peaked above and below the OMZ, Type 3) the distribution peak was above the OMZ, or Type 4) the distribution peak was below the OMZ, and Type 5) there was no obvious peak or pattern in the vertical distribution. Taxa that are located in approximately the same sector of the plot during day and night were non-migrants, such as phaeodarians, Cydippida, *Thalassocalyce inconstans*, *Lilyopsis*, the trachymedusae *Colobonema*, *Haliscera conica* and *Halitrephes*, which all occurred in the core of the OMZ (Type 1). Fishes and euphausiids were found within the OMZ during the day (fishes slightly deeper than euphausiids), but their distributions shifted into the upper layer at night, where concentrations of oxygen, chlorophyll-*a*, and particles were higher (Type 1M; Table S2). Bimodal distribution patterns (Type 2) were found for *Beroe* and for Doliolida. Organisms that were permanently distributed in the UOC (Type 3) were Cestidae, Salpida, Pyrosomida, Gastropoda and the migrating *Resomia* sp (Type 3M). The larvacean *Mesochordaeus*, small narcomedusae, *Atolla*, *Crossota* and *Halicreas*, the ctenophore *Bathocyroe*, Cydippida (small red), and the munnopsid isopods*,* as well as Foraminifera all occurred permanently below the OMZ (Type 4). The distribution peak of decapods and mysids was in the LOC during the day, but shifted to the UL/UOC during the night as a result of vertical migration (Type 4M; Table S2). Example organisms that did not have an obvious distribution pattern in relation to the OMZ (Type 5) were chaetognaths, *Solmundella*, appendicularians, the giant larvacean *Bathochordaeus*, and the polychaete *Poeobiu*s.

## Discussion

The vertical ecological zonation of the water column in the exclusive economic zone of Cape Verde was apparent with distinct but also overlapping distributions for many of the observed animals. These zonation patterns are expected to be influenced by biotic factors such as food availability, and predator avoidance, and also by species-specific responses to abiotic factors, such as oxygen. The correlation between vertical distribution and the oxygen profile allows us to consider and discuss which organisms could potentially benefit or be disadvantaged by OMZ expansion, a phenomenon that is impacting epi-and mesopelagic communities worldwide^7^.

### Taxa absent from the OMZ

Assessment of the potential impact on oceanic food webs starts with the question of which organisms have the most distinct distribution patterns in relation to the OMZ. In our data, the ctenophore *Beroe* and the doliolid tunicates, a predator and a particle-feeder, were discontinuously distributed in the vertical plane, with a gap at OMZ depths. *Beroe* is a widespread ctenophore that feeds on other gelatinous zooplankton and in coastal surveys this genus showed a reduced tolerance of hypoxia compared to medusae and other ctenophores in the same region^52^, which may be related to relatively high respiration rates in the genus (*Beroe ovata*^53^). Several species of *Beroe* occur in the Cape Verde region and additional research is needed to determine if its apparent bimodal distribution is the result of multiple species that are vertically separated above and below the OMZ. Mesopelagic submersible observations from Toyama Bay suggest a bimodal distribution of two *Beroe* species^39^ . In the ETP, doliolids were absent from the OMZ core but were part of distinct communities that inhabited the water column above and below the OMZ^54^. This general distribution pattern fits our observations from the ETNA (Fig. S1), but factors other than oxygen may also be important. For example, contrasting observations in the Arabian Sea found doliolids only in relatively shallow waters that were very low in oxygen^55^. While distribution patterns alone cannot be used to predict species responses, an expanding oxygen minimum zone in the ETNA could potentially shift the vertical distribution of *Beroe* and doliolids, thus reducing their habitat volume. This has been shown to be the case for large pelagic fishes in the ETNA^31^. Such distribution shifts may eventually impact food-web interactions where *Beroe* may be unable to access prey that was previously within its range. Habitat volume reduction and subsequent altered predator prey interactions are serious consequences to be considered since deep-sea food webs may be less resilient than coastal food webs^56^.

The ctenophore family Cestidae and the siphonophore *Resomia* were present above the OMZ at < 300 m off Cape Verde. *Resomia* showed a distinct difference in day and night distribution in the ETNA (Fig. S1). Organisms that occurred below the OMZ included both filter-feeders and predators: *Mesochordaeus*, *Crossota* and *Halicreas*, the ctenophore *Bathocyroe* and a small undescribed red cydippid. *Halicreas* is a species that is known from the mesopelagic and bathypelagic zones in other regions^47,57–60^. In MOCNESS net catches during the same cruise where we collected the video transect data, *Halicreas* occurred in catches that also included the OMZ^61^, but these net samples integrated specimens from 600 to 400 m. Therefore, captured individuals could have come from below the OMZ as well. In Monterey Bay *Halicreas* is found associated with low oxygen levels^62^. Multiple described and undescribed *Bathocyroe* species are known to exist^59^. In North Atlantic Central Water near the Mid-Atlantic Ridge, *Bathocyroe* was among the dominant ctenophores, occurring between 300 and 600 m depth^63^. This distribution is in line with our observations of *Bathocyroe* sp. but they may also be different species.

### Taxa present in the OMZ

Animals that occurred commonly within the OMZ were the siphonophore *Lilyopsis* and the medusae *Colobonema*, *Haliscera conica* and *Halitrephes*. These observations allow us to accept the hypothesis that these gelatinous taxa are inhabitants of the ETNA OMZ. Whether or not these distributions are causally linked to oxygen distribution cannot be determined by observations only, but obviously they are able to tolerate the decreased oxygen levels encountered in this depth layer, as they do not seem to migrate out of it. Low respiration rates have been measured for *Colobonema* and *Halitrephes maasi*^33^ and *Colobonema* was also found within the OMZ during net catches coincident with the present PELAGIOS data^61^. In two locations in the tropical western Pacific, *Haliscera conica* has a mean vertical distribution depth of about 700 m, (oxygen levels of around 111.65 µmol kg^−1^ and less than 89.32 µmol kg^−1^)^64^.

While oxygen may limit the distribution of some species, we also show that other organisms were widely distributed and that general, non-specific distribution patterns were observed frequently in our data. An example of an organism that was observed in the OMZ but was not restricted to the OMZ is *T. inconstans*. This ctenophore is known from Monterey Bay and its feeding behavior has been described from ROV observations. The species is able to capture relatively large prey (euphausiids) using the sticky mucus on the inside of the lobes^65^. The vertical distribution of *T. inconstans* in the ETNA overlaps with that of euphausiids, and these ctenophores may trap vertically migrating krill as has been suggested for *Kiyohimea usagi*^66^. For euphausiids, species-specific hypoxia tolerance has been determined by respiration experiments^67,68^, and was around 20 µmol kg^−1^ (at 11 °C) in the ETNA^35,67^, which is below the lowest oxygen concentration encountered during our transects. The giant larvacean *Bathochordaeus* is another genus that is distributed in the ETNA OMZ, as well as at other depths.

Narcomedusae of the genus *Solmissus* were the most abundant medusae encountered during our transects. They were widely distributed throughout the water column, and we likely encountered multiple species on our transects. These results agree with net catches during the same cruise^69^ where *Solmissus* was also found in the ETNA OMZ as well as elsewhere in the water column^61^. In the eastern Pacific *Solmissus* also has a wide vertical range, including the OMZ between 600 and 800 m depth^62,70^. Mediterranean *S. albescens* have a relatively low metabolic rate^71^ and if similar physiological traits exist in *Solmissus* spp. from the ETNA, this may explain the ability to inhabit the ETNA OMZ. During our surveys, specimens were often observed with their tentacles up and down, which is apparently a feeding position to capture other gelatinous zooplankton^43,72^. The polychaete *Poeobius* sp. also showed a very wide vertical distribution. Suggestions for extreme tolerance of low-oxygen conditions inside mesoscale eddies has been reported for *Poeobius* in the eastern Atlantic^22^, and was attributed to low respiration rates, as measured for this genus of pelagic polychaetes in the Pacific^73^.

Another group of frequently observed and widely distributed organisms included Rhizaria, a eukaryotic super kingdom that are among the dominant planktonic members in the oceans worldwide^74^. The distributions that we reconstructed did not reveal a specific pattern, although the peak of Phaeodaria was inside the OMZ. However, the patterns should be considered with care since, as is the case for other taxa we report on, they likely include multiple species, and most are probably not well identifiable with the given image resolution.

### Diel vertical migration

Euphausiids and fishes each showed distinct differences in their day and night distributions, occurring mostly in shallow layers at night and residing in the OMZ during the day. The data on both fishes and euphausiids observed by PELAGIOS are pooled observations and consist of a variety of species. In the Pacific, myctophid fishes inhabit the upper oxycline and the upper part of the oxygen minimum zones during the day, probably to avoid predators^1,11,75^. This predator avoidance also drives the downward migration and the depth of penetration into the mesopelagic zone by these actively swimming fishes, but the migration depth is likely impacted by the (in)ability to cope with low oxygen levels. In the Pacific, this results locally in the presence of the deep scattering layer (DSL) just above the hypoxic zone^76^. Deeper DSL may be present in the South Pacific and North Atlantic at depths of 500–1000 m^9^. The strong OMZ at 600–800 m in the Pacific off California may explain the absence of a deeper DSL consisting of mesopelagic zooplankton, myctophid fishes and other micronekton^77^. In the Red Sea, a region with a strong OMZ and high water temperatures, four distinct scattering layers were found, of which two were present above the OMZ, one was present inside the OMZ and another layer was found below the OMZ^78^. A global analysis shows that daytime depth of the DSL is coupled to a “common optical depth layer” and that oxygen and light can explain the shallow occurrence of DSL in hypoxic regions^79^.

Other migrating organisms included decapods and mysids, the coronate medusa *Atolla* and *Bargmannia* siphonophores, but the daytime distribution of these organisms differed from the fishes and euphausiids in that the distribution peak was below the OMZ. For some gelatinous predators, it appears that they follow their food source rather than hide from predation, given their low nutritional value combined with effective defense (cnidocytes) and camouflage (transparency) mechanisms could, in principle, enable them to stay in the surface layer provided that temperature is not an issue. In Monterey Bay, *Atolla* is also found below the OMZ^80^ and the vertical migration behavior observed here is known for the genus *Atolla* in other regions^81,82^. Little data is available on *Bargmannia* siphonophores and while our data suggest that this genus migrates vertically, as also observed in the western Pacific^83^, more observations are needed to confirm this behaviour in the ETNA. The observed munnopids are likely too few to draw conclusions on migration. For the cephalopods, chaetognaths, munnopsids and appendicularians it is not clear if the observed pattern truly represents vertical migration. During the day smaller gelatinous taxa like larvaceans and chaetognaths may be harder to observe due to reduced contrast in shallower waters while cephalopods may avoid the PELAGIOS system in shallower waters. Similar problems have been discussed for horizontal video surveys in the Pacific^84^. The absence of a significant difference in weighted mean depth in day and night distribution of pyrosomes and salps is likely due to low sample size (both transects with occurrence and total number of specimens observed) as the PCA does suggest migration behaviour in both taxa, with the daytime depth in the UOC and the nighttime depth in the UL.

### In situ observations to reveal oceanic biodiversity

Our data illustrate how in situ observations can increase the understanding of local pelagic biodiversity. The video transects revealed more gelatinous taxa than were captured in nets during the same cruise^50,69^. We report here only the species that were abundant enough to enable reconstruction of their vertical distribution patterns. There were numerous “rare” gelatinous species encountered during our video transects but these will be the subject of a separate study. For the taxa we consider here, several were first records of occurrence in the region. For example, *Lilyopsis* was only known from the North Atlantic and the Canary islands, Mediterranean and Pacific^85^. The giant larvacean *Mesochordaeus* was, to our knowledge, here observed for the first time in the eastern Atlantic although it is known to occur in the Bahamas region^86,87^. These first records follow already published new records observed by in situ observations from the PELAGIOS^22,66^.

## Conclusion and outlook

We provide high resolution baseline data on vertical distribution patterns of pelagic fauna in the Cape Verde region of the eastern Atlantic. These data contribute to the essential ocean variable “zooplankton biomass and biodiversity”, which is still relatively poorly documented, in particular in the Cape Verde region despite it being a hotspot of oxygen minimum expansion^88^. Due to its ecological relevance, the upper oxycline has been recognized as a useful proxy for ecosystem monitoring objectives in eastern boundary systems close to oxygen minimum zones^89^, and here we link this parameter with faunal distributions. Future efforts should focus on increasing the taxonomic resolution of the in situ observations. Faunal presence in the OMZ may be related to the fact that the ETNA OMZ is relatively weak. Interactions such as prey and particle distribution (for filter feeding), light and temperature, may be other important drivers of vertical distributions. In addition, some of the taxonomic groups that we pooled, likely consist of multiple species, and the broad distribution may actually represent several species-specific distributions together. One step forward would be to perform ROV video surveys in combination with faunal collections for morphological and genetic identification. Expanding oxygen minimum zones will change the trophic interactions of the pelagic ecosystem which will impact the efficiency of the biological carbon pump and hence carbon cycling and sequestration. An interdisciplinary approach that combines biodiversity research, physiology and biological, physical and chemical oceanography is needed to disentangle the multitude of factors that drive animal distribution in a rapidly changing ocean.

## Methods

During the research cruise MSM49 of R/V *Maria S. Merian* in November–December 2015 in waters around the Archipelago of the Republic of Cape Verde^90^, we performed horizontal video transects with the pelagic in situ observation system (PELAGIOS)^50^. PELAGIOS collects HD video (1Cam Alpha, SubC Imaging, Sony HDR-CX760V recorder, MTS files, 50 frames/s) as well as CTDO data. However, we only monitored depth with the PELAGIOS CTD during the tows and used data from vertical CTD casts (see below) for presentation of hydrographic conditions here because the latter CTD was calibrated with discrete Niskin bottle samples. PELAGIOS was towed at approximately 1 knot (0.51 m/s) for 11 or 22 min, but in some cases up to 36 min, at desired depths from 50 m down to 1000 m (Table S1). In total, 13 PELAGIOS deployments are considered here, which took place at seven stations (Fig. 1). At six of the stations, both day and night deployments were made at similar depths (50–1000 m) to investigate diel vertical migration (Table S1). During MSM49 a bar with white marks (spaced 5 cm from each other) was placed 1 m in front of the camera which allowed estimation of the size of encountered organisms when they were lined up with the bar.

CTD-profiles used to define the study area were recorded with the ship’s CTD/rosette system consisting of an SBE 911 plus CTD with double conductivity, temperature and dissolved oxygen sensors (Sea-Bird Scientific), as well as a combined fluorescence/turbidity sensor (Wetlabs) and a PAR-sensor (Biospherical Instruments QSP2350). Oxygen sensor data were calibrated against 173 discrete Winkler titration samples obtained during the cruise. An Underwater Vision Profiler 5 (UVP5 serial number #000^91^) was mounted on the CTD/rosette frame. Particles > 64 µm ESD were sized and enumerated. For objects > 500 µm ESD, images were saved, sorted into taxonomic categories and archived using EcoTaxa (https://ecotaxa.obs-vlfr.fr, Picheral et al. 2017). Particle and copepod abundances were exported from the EcoPart module for further analysis in 5 m depth bins and 25–50 m depth bins, respectively.

The collected video (> 40 h) was analyzed using the Video Annotation and Reference System (“VARS”, MBARI^92^). This analysis resulted in distribution data for 46 taxonomic groups (Table S2) including ctenophores, medusae, siphonophores, polychaetes, fishes, crustaceans and tunicates (see also example framegrabs at https://doi.pangaea.de/10.1594/PANGAEA.919335). The data collected at MSM49 station Senghor NW was also used in the methodology publication about PELAGIOS by Hoving et al.^50^. In that study the day and night distribution data of larger taxonomic groups was presented, including fishes, chaetognaths, decapods, mysids and euphausiids, ctenophores, medusae, siphonophores, salps, doliolids and larvaceans, as well as a list of taxa observed during MSM49, as identified to the lowest possible taxon. We use the data from Senghor NW here to study the vertical distributions in more detail, also in comparison with the other stations.

Organisms that were observed in-between transects while PELAGIOS was ascending or descending were annotated but not considered in our data analysis. After the annotation analysis of the video material, the annotations were matched with the depth data and classified with the corresponding transects. Each annotated organism was linked to the targeted depth of the video transect, which sometimes deviated from the exact acquisition depth since the camera system moved up and down during towing, depending on the sea state. This deviation was mostly less than or around 5 m, but several of the deeper transects had larger deviations of up to 25 m (Fig. S2). The target depths are used in the figures. The taxa observed during the individual transects were summarized and then divided by the duration of each corresponding transect to obtain the relative abundance of taxa in individuals per time unit. To calculate the number of individuals per volume (1000 m^3^), we used a conversion factor that was calculated based on simultaneous deployments of the PELAGIOS and a UVP5 together^50^. The conversion factor is based on pelagic worms of the genus *Poeobius* (0.8–1.5 cm in length) which are poor swimmers and which were observed clearly by both instruments and was used to estimate the volume scanned by PELAGIOS per time unit. Abundances were then obtained by dividing the number of individuals counted on the transect by the estimated volume scanned over the duration of the transect, and scaling to 1000 cubic metres. For further details about this conversion from individuals counted per minute to individuals per volume we refer to the PELAGIOS methodology publication^50^. This conversion factor may be suboptimal when considering larger animals since a larger organism may be seen from further away^93^, but it was our only frame of reference for estimating volume.

Graphs were produced in R Studio version 3.6.1 and Python (using matplotlib and basemap).

The annotated fauna was identified to the lowest taxon possible, resulting in 46 different groups. Unicellular zooplankton large enough to be detected with PELAGIOS and with characteristic shapes were annotated, most were not. The annotated unicellular zooplankton was divided into Foraminifera, characterized by their numerous pseudopods^94^ and phaeodarians of the family Coelodendridae which were distinctive by their four long ‘styles’ called appendages^95^ that were always observed in a downward orientation. Only Scyphozoans of the order Coronatae were observed, with *Atolla* being the most commonly observed genus. *Atolla* specimens were identified by their non-transparent reddish color, their disc-like umbrella and thick tentacles, with the elongated hypertrophied tentacle visible in many cases^96^. Coronates that were lacking a hypertrophied tentacle and had an umbrella that was not flat and disc-like were grouped into ‘other_Coronatae’.

Observed Hydrozoa were grouped into the three orders Trachymedusae, Narcomedusae and Siphonophorae. Identified genera of the order Trachymedusae were *Colobonema*, *Crossota*, *Halicreas*, *Haliscera* and *Halitrephes*. Both *Colobonema* and *Crossota* showed a similar bell-like umbrella. *Colobonema* was transparent with tentacles that had 3–4 times the length of the umbrella. *Crossota* appeared only partly transparent with some red color in the umbrella and had short white tentacles. Of the family Halicreatidae, *Halicreas* was identified by the small apical projection and the presence of eight pronounced radial canals. *Haliscera* was identified by its cone-shaped umbrella and *Halitrephes* was identified by its thick, rounded umbrella and the numerous tentacles and the presence of more than 8 radial canals^97^. Narcomedusae observed on the video could be identified as the genera *Solmissus* or *Solmundella*, or were otherwise grouped into the ‘small_Narcomedusae’. *Solmundella* was identified by the presence of only two tentacles pointing towards the swimming direction. Other Narcomedusae smaller than *Solmissus* with four tentacles, resembling the habitus of the genera *Aegina* and *Solmundaegina* as described by^44^ were combined into the group ‘small_Narcomedusae’. The Siphonophorae included the two suborders Physonectae and Calycophorae. Small fast-moving calycophoran siphonophores with a pointed anterior nectophore were identified as members of the family Diphyidae. The two prayid genera *Praya* and *Lilyopsis* could be identified from the video observations. *Praya* specimens were distinctive by their large size and bright yellow gastrozooids^98^. *Lilyopsis* showed green fluorescence which was strongest at its posterior end^85^. The two physonect siphonophore genera *Bargmannia* and *Resomia* were identified based on resemblance with colonies observed by ROV in Monterey Bay (MBARI Deep-Sea Guide). The observed *Bargamnnia* colonies had a relatively long nectosome and they were dragging their tentacles behind them. Lobate ctenophores of the genus *Bathocyroe* were distinguished from other Lobata by their red pigmented stomodaeum in combination with their big lobes^59^. Appendicularians of considerable size were identified as either one of the genera *Bathochordaeus* or *Mesochordaeus*. Instead of the actual animal, we based our identification on the appearance of the filtering houses. Specimens of the genus *Bathochordaeus* were the largest appendicularians we observed. Their inner filtering house was small compared to the extensive outer mucous house surrounding it^99^. The *Mesochordaeus* houses were characterized by their very large inner filter that was surrounded by only a thin outer mucous layer^87^. Doliolida were only observed and identified as colonies of a non-reproductive nurse which dragged multiple trophozooids.

Example framegrabs of the dominant annotated fauna can be found at https://doi.pangaea.de/10.1594/PANGAEA.919335.

For the 46 taxonomic groups, the weighted mean depths (WMD) were calculated based on the formula by Latasa et al.^100^ which summarizes the product of abundances and individual depths and divides it by the summarized abundances (Table S2) The two taxa *Beroe* and Doliolida showed bimodal distributions and were therefore split into two different groups to make WMD calculations meaningful. The night and day distributions were compared by conducting paired t-tests of the WMD of the day and night hauls (Table S2).

To compare the daytime and nighttime community composition across samples, a principal component analysis (PCA) was conducted on abundance and environmental data (T, O_2_, Chlorophyll-*a* and particle concentration) that were normalized to range between 0 and 1.

### Ethics approval

No ethical approval was required. We did not collect organisms for this study.

## Supplementary information

Supplementary Information

## Data Availability

CTD data are available at https://doi.org/10.1594/PANGAEA.910346, UVP5 data are available at https://ecotaxa.obs-vlfr.fr/part/ (project #588, UVP5 Geomar 2015 msm049), PELAGIOS data and example images of annotated fauna are available at PANGAEA (https://doi.pangaea.de/10.1594/PANGAEA.919335).
