# Supplementary material for: In situ observations show vertical community structure of pelagic fauna in the eastern tropical North Atlantic off Cape Verde
Source: Sci Rep. 2020 Dec 11;10:21798. doi: 10.1038/s41598-020-78255-9 (PMC7733502; doi:10.1038/s41598-020-78255-9)
Supplement: Supplementary file 1 — Supplementary Information [file 41598_2020_78255_MOESM1_ESM.pdf]

In situ observations show vertical community structure of pelagic fauna in the eastern tropical North Atlantic off Cape Verde

Hoving, H.J.T.[1*], Neitzel, P.[1], Hauss, H.[1,2], Christiansen, S.[1,3], Kiko, R.[1,4], Robison, B.H.[5], Silva, P[6], & Körtzinger, A.[1,2]

[1]GEOMAR Helmholtz Centre for Ocean Research Kiel, Düsternbrooker Weg 20, 24105 Kiel, Germany.

[2]Christian Albrecht University Kiel, Christian-Albrechts-Platz 4, 24118 Kiel, Germany

[3]University of Oslo, Blindernveien 31, 0371 Oslo, Norway

[4]Laboratoire d'Océanographie de Villefranche, Sorbonne Université, Villefranche-sur-Mer, France

[5]Monterey Bay Aquarium Research Institute, Sandholtroad 7700, Moss Landing, USA

[6]Ocean Science Centre Mindelo & Instituto do Mar (IMAR), Cova de Inglesa, C.P. 132, Mindelo, São Vicente, Rep. of Cabo Verde

*Correspondence to: H.J.T. Hoving, GEOMAR Helmholtz Centre for Ocean Research Kiel, Düsternbrooker Weg 20, 24105 Kiel, Germany.

hhoving@geomar.de; +49 4316004566

Keywords: deep sea, in situ observations, vertical distribution, gelatinous zooplankton, micronekton, oxygen minimum zone, mesopelagic zone

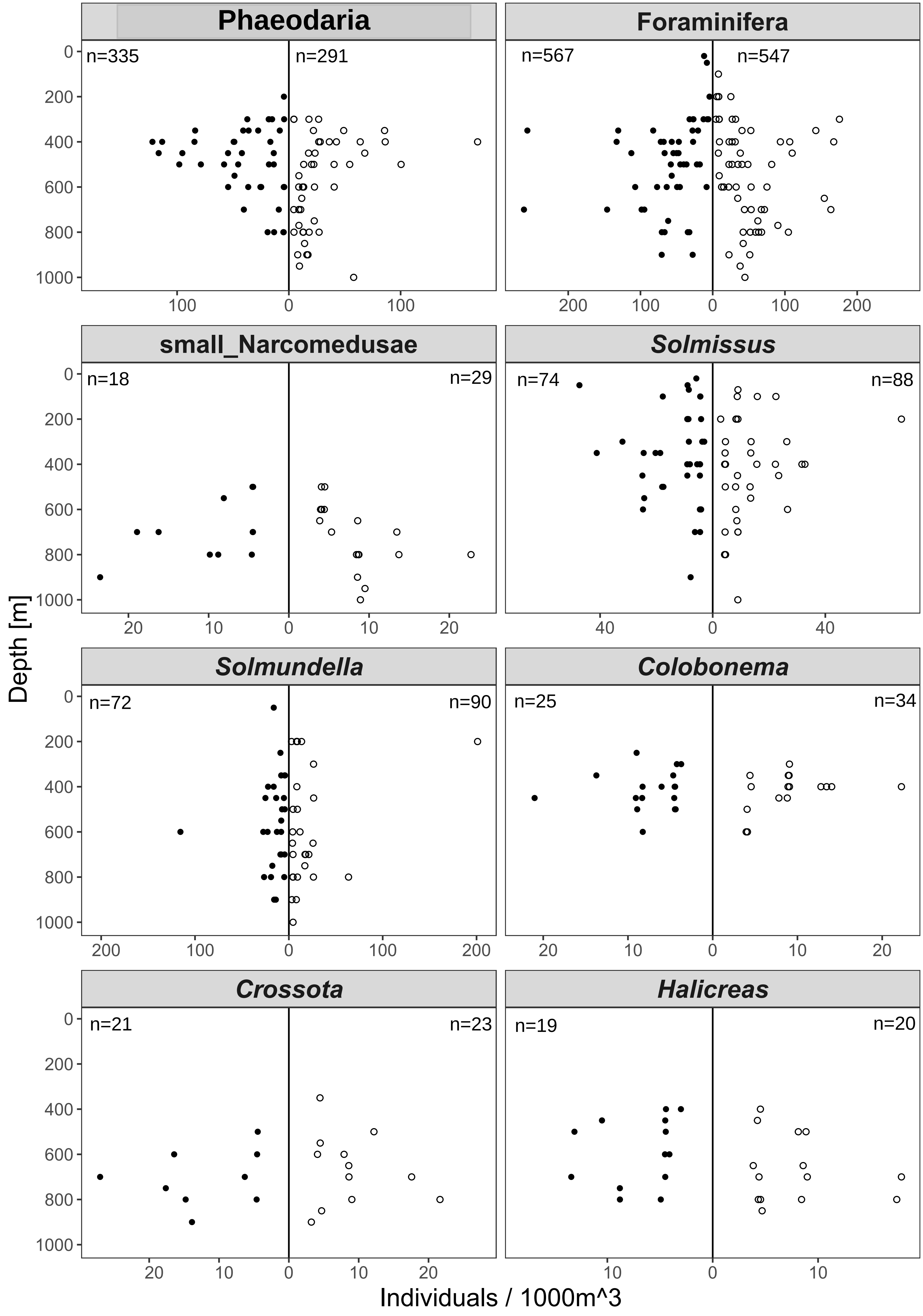

Phaeodaria
n=335
n=291
Foraminifera
n=567
n=547
small_Narcomedusae
n=18
n=29
Solmissus
n=74
n=88
Solmundella
n=72
n=90
Colobonema
n=25
n=34
Crossota
n=21
n=23
Halicreas
n=19
n=20
Depth [m]
Individuals / 1000m^3

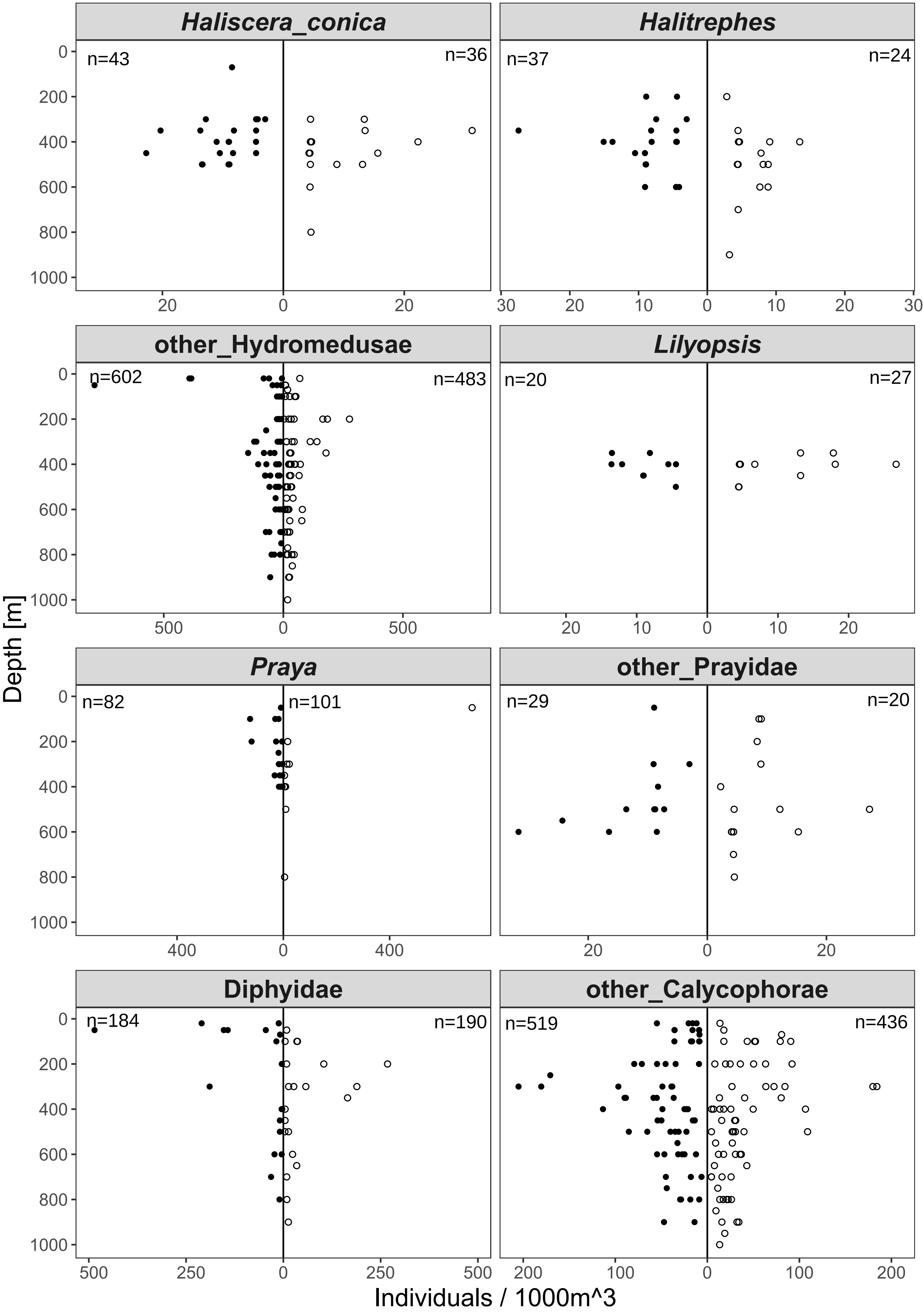
Haliscera_conica
n=43
n=36
Halitrephes
n=37
n=24
other_Hydromedusae
n=602
n=483
Lilyopsis
n=20
n=27
Praya
n=82
n=101
other_Prayidae
n=29
n=20
Diphyidae
n=184
n=190
other_Calycophorae
n=519
n=436
Depth [m]
Individuals / 1000m^3

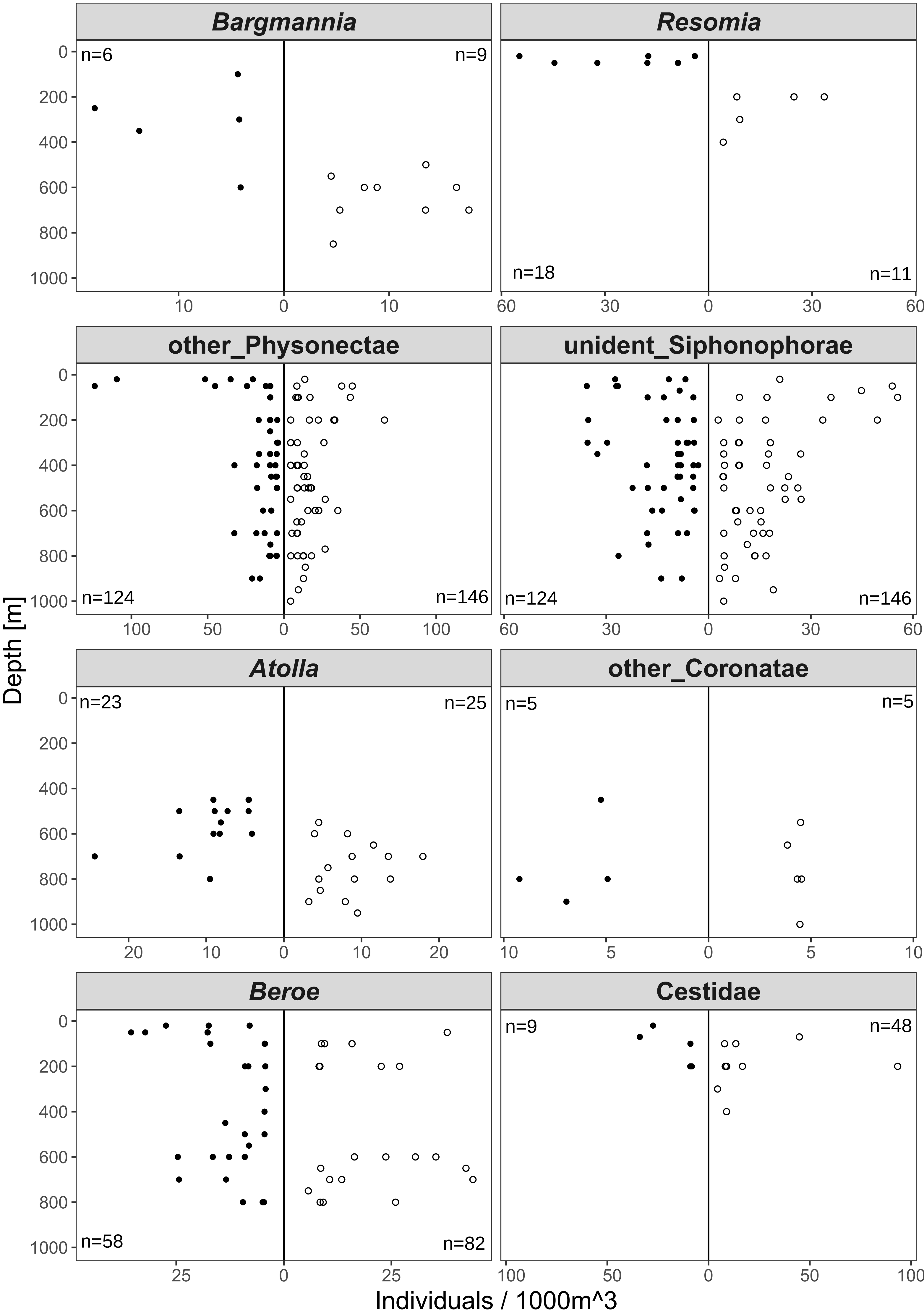
Bargmannia
n=6
n=9
Resomia
n=18
n=11
other_Physonectae
n=124
n=146
unident_Siphonophorae
n=124
n=146
Atolla
n=23
n=25
other_Coronatae
n=5
n=5
Beroe
n=58
n=82
Cestidae
n=9
n=48
Depth [m]
Individuals / 1000m^3

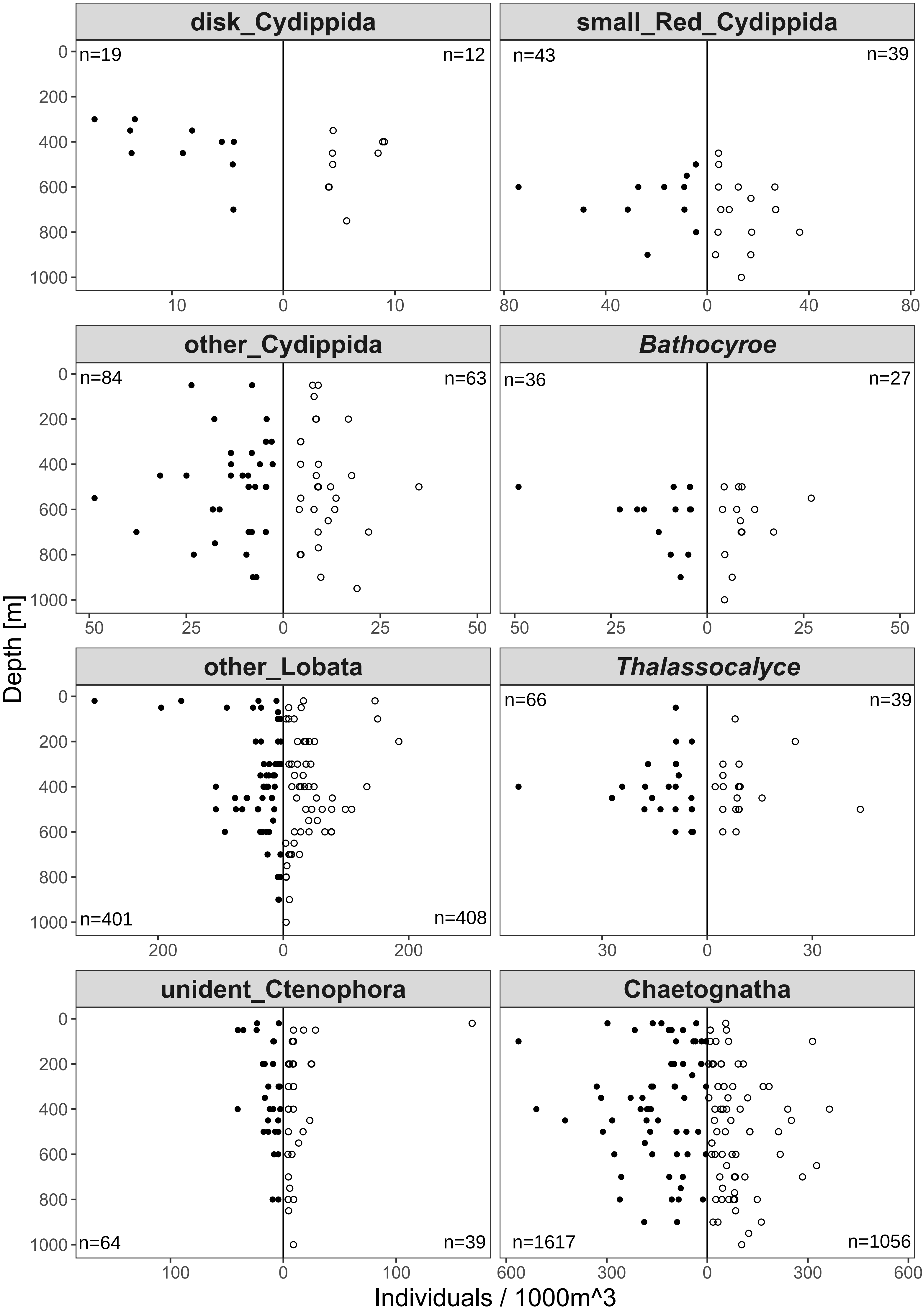

disk_Cydippida
n=19
n=12
small_Red_Cydippida
n=43
n=39
other_Cydippida
n=84
n=63
Bathocyroe
n=36
n=27
other_Lobata
n=401
n=408
Thalassocalyce
n=66
n=39
unident_Ctenophora
n=64
n=39
Chaetognatha
n=1617
n=1056
Depth [m]
Individuals / 1000m^3

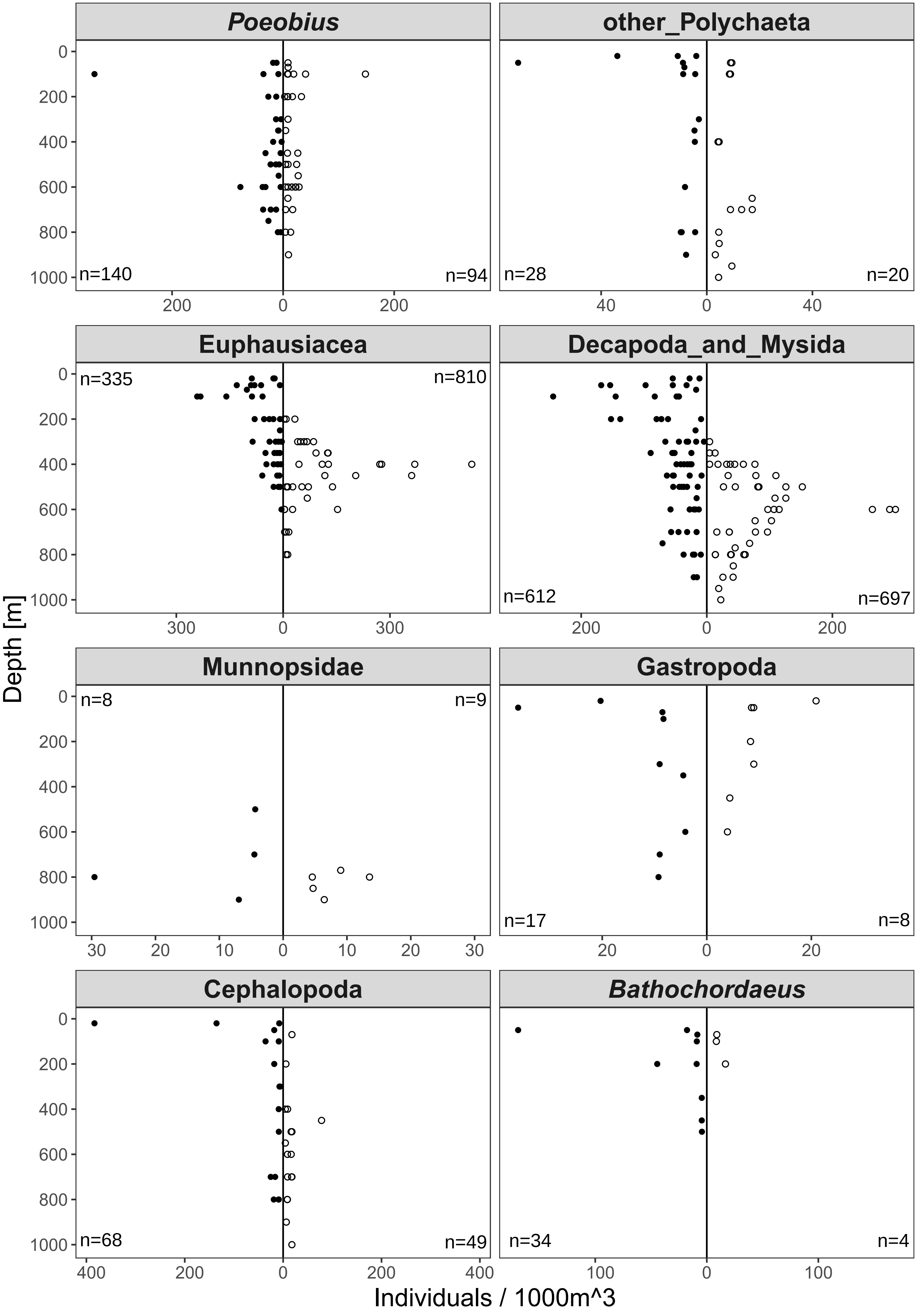

Poeobius
other_Polychaeta
Euphausiacea
Decapoda_and_Mysida
Munnopsidae
Gastropoda
Cephalopoda
Bathochordaeus
n=140
n=94
n=28
n=20
n=335
n=810
n=612
n=697
n=8
n=9
n=17
n=8
n=68
n=49
n=34
n=4
Depth [m]
Individuals / 1000m^3

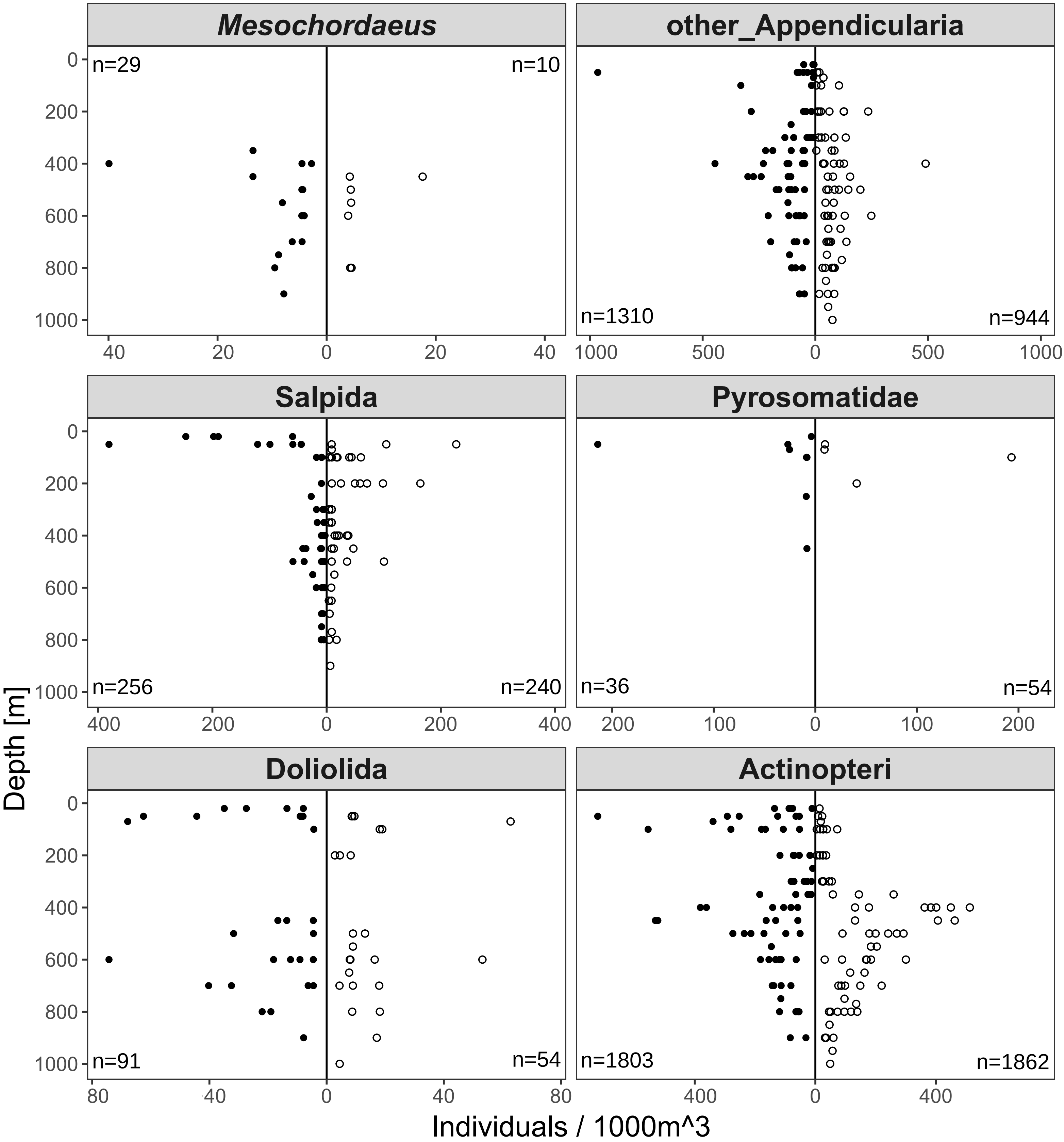

Figure S1: Distribution profiles of all taxonomic groups encountered during pelagic video transects with PELAGIOS during MSM49 in the Cape Verde region.

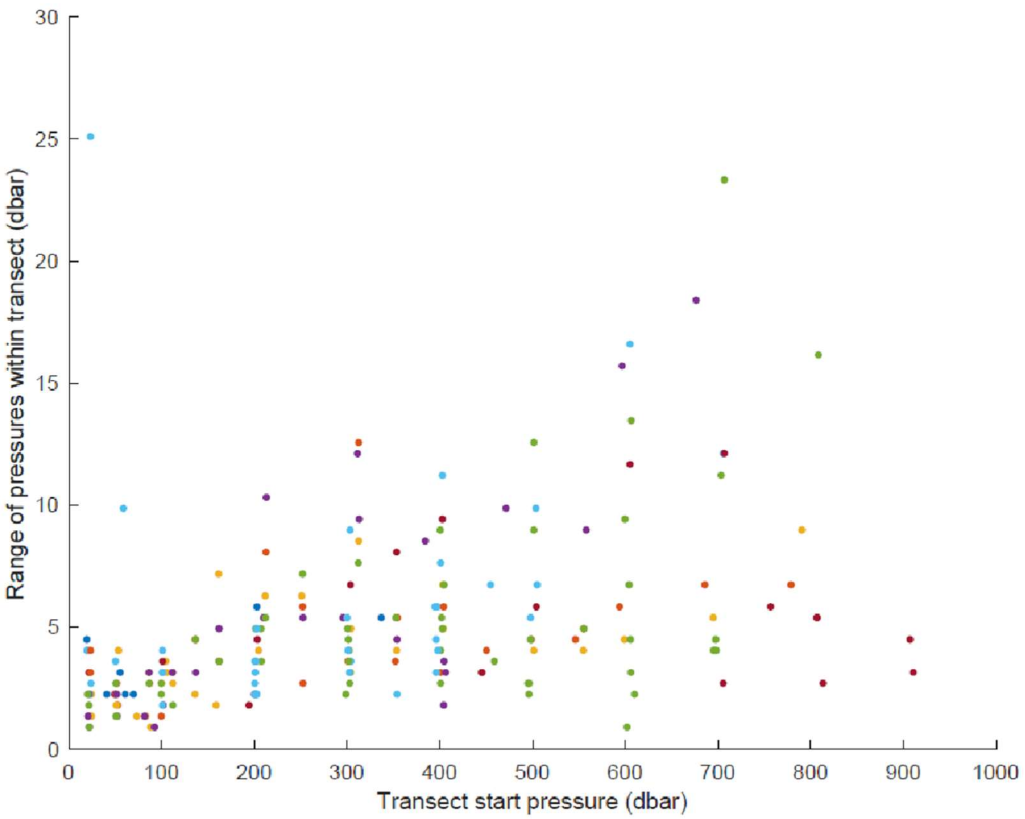

Fig. S2: Absolute values of deviation from target depth (y-axis) in relation to target depth (x-axis) of PELAGIOS horizontal video transects.

Table S1: Date, depth, length and station information for the pelagic video transects undertaken during cruise MSM49 in November, December 2015 in Cape Verdean waters.

Station		CVOO	CVOO	CVN	CVN	Senghor Ref	Senghor Ref	Senghor NW	Senghor NW	Senghor SE	Senghor SE	CVS1	CVS1	CVS2	CVS2
Haul		Night	Day	Day	Night	Day	Night	Day	Night	Day	Night	Day	Night	Day	night
Latitude<br><br>Longitude		17° 39.67' N<br><br>24° 13.58' W	17° 35.06' N<br><br>24° 17.07' W	17° 59.99' N<br><br>23° 00.02' W		18° 05.01' N<br><br>22° 00.00' W	18° 04.98' N<br><br>22° 00.02' W	17° 14.18' N<br><br>22° 00.66' W	17° 14.22' N<br><br>22° 00.73' W	17° 09.16' N<br><br>21° 54.68' W	17° 09.72' N<br><br>21° 55.31' W	12° 01.21' N<br><br>20° 57.24' W	12° 00.85' N<br><br>20° 57.61' W	12° 00.85' N<br><br>22° 57.63' W	12° 00.85' N<br><br>22° 57.63' W
start (UTC)<br><br>end (UTC)		2:22:43<br><br>6:27:53	12:44:35<br><br>15:21:23	14:33:08<br><br>19:07:42		11:38:02<br><br>17:34:14	3:03:06<br><br>7:37:40	13:30:29<br><br>18:05:02	23:10:37<br><br>5:52:47	13:10:47<br><br>17:45:07	2:48:41<br><br>7:09:06	12:11:01<br><br>17:31:58	20:05:20<br><br>0:51:43	11:02:46<br><br>16:58:35	21:00:36<br><br>2:09:10
Sum transect duration [h:min:sec]		2:48:58	1:59:07	3:25:27		3:14:46	3:34:35	3:07:00	4:51:53	3:19:47	2:58:05	3:59:26	3:36:09	4:08:54	3:44.11
Transect depths [m] and duration [min:sec]	20	1:05	4:47	7:16			17:11		14:14		25:10	3:07	9:06		14:46
	50	8:27	10:32	11:14		11:09	11:17	11:25	11:18	13:13	12:25	11:08	11:12	11:41	11:05
	70											11:09	11:47		
	100	11:17	12:37	11:02		11:40	11:43	10:32	22:29	11:32	12:04	22:16	22:52	11:28	11:11
	200	10:59	11:56	11:09		12:07	11:04	12:11	23:14	35:20	24:16	22:07	22:40	11:57	11:15
	250	11:08													
	300	15:36	11:24	22:01		22:05	22:32		33:20	22:24	23:28	22:31	26:54	22:14	22:10
	350	12:14					7:17	22:44	24:37			22:11	21:28	22:25	22:13
	400	12:21	11:15	44:39		23:29	22:16	21:22	33:09	22:27	36:15	22:02	22:07	22:05	22:33
	450	12:01		12:48			22:03		19:02			23:33	22:02	22:43	22:12
	500	13:48	11:01	22:21		22:54	22:03		22:52	24:37	22:27	22:14	22:26	22:35	22:17
	550	12:20		7:22				22:15							
	600	12:07	11:17	13:05		24:39	22:05		24:21	24:23	22:00	22:38	23:35	25:18	21:59
	650							25:59						11:39	
	700	12:19	11:10	18:47		22:17	22:21		15:51	22:45				11:37	22:12
	750							17:36	11:21						
	770							11:05							
	800	10:31	11:27	23:43		22:01	22:43		21:40	23:06		21:52		22:10	20:18
	850							21:19							
	900	12:45	11:41						14:25			12:38		31:02	
	950							10:32							
	1000					22:25									

Table S2: Taxonomic groups considered in this study, as well as their weighted mean distribution depth (m) during day (D) and night (N) and the type of distribution (Type 1-5) in relation to the oxygen minimum zone (OMZ). When the p-value is lower than 0.05 (as indicated by *), there is a significant difference between the day and night distribution and hence vertical migration (indicated by "M" after the distribution type).

Taxon	time	Station							n	Type	p-value
		CVOO	CVN	Senghor Ref	Senghor NW	Senghor SE	CVS1	CVS2			
Phaeodaria	N	469.9	/	507	445.3	515.1	423.3	427	335	1	0.08
	D	471.5	513.5	616.9	606.3	530.1	426.9	494.9	291		
Foraminifera	N	570.7	/	540.4	630.7	427.1	416.1	506.6	567	4	0.07
	D	522.4	609.9	606	702.7	604.6	537.3	541.7	547		
Small Narcomedusae	N	772.9	/	698.5	719.6	500	NA	768.6	18	4	0.26
	D	849.5	697.2	792.4	863.5	678.8	751.3	634.2	29		
Solmissus	N	340.7	/	327.2	401.3	307.7	270	434.1	74	1	0.29
	D	311.6	402.1	570.1	435.6	470	242	395.3	88		
Solmundella	N	582.8	/	707	685.5	50	600	530	72	5	0.88
	D	280.2	572.7	748	581.7	504.2	560.1	575	90		
Colobonema	N	428.1	/	391.5	447.4	421.8	403.9	400	25	1	0.23
	D	400	418.4	358.5	388.1	440.3	366.7	425.5	34		
Crossota	N	NA	/	NA	742	550.5	NA	735.4	21	4	0.88
	D	NA	NA	NA	703.2	687.7	NA	670.5	23		
Halicreas	N	NA	/	716	545.2	500	NA	593.1	19	4	0.29
	D	749.4	800	733.6	759.9	629.3	450	538	20		
Haliscera conica	N	356.5	/	424.9	381.6	431.9	312.9	433.2	43	1	0.56
	D	NA	450	500	400	362.4	471.9	383.1	36		
Halitrephes	N	374.9	/	419.6	432	466.1	371	393.8	37	1	0.2
	D	NA	470.9	601.4	400	422.5	498.7	507.9	24		
other Hydromedusae	N	458.5	/	417.6	295	75.7	117.7	258.8	602	5	0.13
	D	322.7	352.2	392.4	532.5	489.5	453.4	382.7	483		

Lilyopsis	N	NA	/	NA	379.9	400	433.2	391.7	20	1	0.41
	D	NA	440	NA	363.1	400	400	403.9	27		
Praya	N	254.4	/	358.4	306.5	232.7	130.6	96.6	82	3	0.2
	D	500	NA	268.1	375.8	316.6	427.8	52.2	101		
other Prayidae	N	559.6	/	570	448.9	451.8	317.9	300	29	5	0.47
	D	500	412.7	705.6	NA	425.8	549.6	251.8	20		
Diphyidae	N	50	/	20	NA	57.1	66.1	216.8	184	**3M**	**0.01***
	D	200	100	300	NA	326.3	232	314.1	190		
other Calycophorae	N	372.7	/	401.7	406.7	310.5	324	386.5	519	1	0.31
	D	376.7	334.5	385.7	637.7	394	287.9	394.9	436		
Bargmannia	N	250	/	350	600	300	100	NA	6	**4M**	**<0.01***
	D	600	641.1	700	703.2	651.7	500	NA	19		
Resomia	N	NA	/	35.1	24.2	46.7	50	NA	18	3M	0.06
	D	200	NA	246.2	200	NA	NA	NA	11		
other Physonectae	N	536.9	/	125.9	287.9	105.5	258.4	281.7	124	5	0.24
	D	315.8	475.4	361.3	532.8	486.7	222.9	383.8	146		
unidentified Siphonophorae	N	548.6	/	360.5	432.5	373.9	268.8	313.3	124	5	0.7
	D	247.3	511.5	385.7	640.3	521.9	199.8	495.8	152		
Atolla	N	655.8	/	586.9	600	500	450	487.5	23	**4M**	**0.01***
	D	700	NA	740.3	758.8	651.7	836.6	759.9	25		
other Coronatae	N	NA	/	NA	746.5	NA	NA	800	5	4	NA
	D	NA	NA	899.1	596.1	800	NA	NA	5		
Beroe above 300 m	N	50	/	77.5	50.9	89.1	100	NA	26	2	0.08
	D	82.9	200	NA	146.4	200	NA	149	22		
Beroe below 300 m	N	667.2	/	549.6	631.6	600	600	657	32	2	0.06
	D	NA	655.4	700	661.8	711.1	641.1	NA	60		
Cestidae	N	NA	/	200	20	200	70	100	9	3	0.2
	D	229.3	200	200	NA	204.6	76.9	NA	48		
Cydippida TypeA	N	350	/	409.2	NA	324.6	NA	433.5	19	1	0.23
	D	NA	NA	600	750	463	450	419.7	12		
small red Cydippida	N	674.5	/	652.8	NA	NA	600	620	43	4	0.33
	D	728.5	744.2	772.4	NA	NA	549.6	660.6	39		

other Cydippida	N	485.7	/	497.3	638	445.1	474.8	372	84	5	0.57
	D	350	550	534.5	793.7	482.5	408.8	511.6	63		
Bathocyroe	N	707.1	/	550	655.2	531.7	548.8	642.6	36	4	0.53
	D	NA	600	741.1	550	602.3	500	712.8	27		
other Lobata	N	394.5	/	232	245.8	305	417.7	348.8	401	5	0.23
	D	213.9	478	443.8	515.5	471.3	398.9	453.1	408		
Thalassocalyce	N	387.4	/	422.1	414.7	429	384.4	341.4	66	1	0.81
	D	313.5	443.7	465.6	400	467.6	483.5	288.5	39		
unidentified	N	340.1	/	197.1	444.5	191.7	413.8	NA	64	5	0.82
Ctenophora	D	59.5	423.7	458.2	376.4	293.7	288.3	NA	39		
Chaetognatha	N	470.6	/	377.6	412.9	266.1	97.8	358.3	1617	**5M**	**0.04***
	D	426.1	377	550.8	673	544.7	671.9	498.2	1056		
Poeobius	N	284.9	/	536.3	643.9	488.4	382.5	164.3	140	5	0.32
	D	285.8	494.9	312.7	110.6	555.1	256.7	309.5	94		
other	N	766.2	/	800	300	20	79.1	160.6	28	5	0.43
Polychaeta	D	365.5	NA	488	788.7	700	75	694.3	20		
Euphausiacea	N	138.4	/	300	169.6	163.4	140	182.2	**335**	**1M**	**<0.01***
	D	474.3	499.1	370.7	387.3	410.4	403.2	409.5	810		
Decapoda and Mysida	N	301.6	/	284.2	304.7	250.3	315	316	612	**4M**	**<0.01***
	D	630.8	537.6	601.3	651.1	611	542.4	600.3	697		
Munnopsidae	N	NA	/	NA	745.3	NA	NA	786.8	8	**4M**	**0.04***
	D	NA	NA	NA	797.4	NA	800	832.3	9		
Gastropoda	N	NA	/	NA	738.4	100	70	162.1	17	5	0.62
	D	20	NA	NA	NA	NA	50	267	8		
Cephalopoda	N	753.9	/	397.4	90.1	20	191.9	38	68	**4M**	**0.03***
	D	700	483.3	899.6	550	554.1	284.2	754.5	49		
Bathochordaeus	N	200	/	50	500	50	70	214.5	34	5	0.61
	D	NA	NA	100	NA	NA	70	200	4		
Mesochordaeus	N	751.2	/	500	664.2	461.8	400	433.7	29	4	0.3
	D	NA	NA	NA	550	800	631.5	481.3	10		

other Appendicularia	N	453	/	483.6	572.7	305.6	361.6	288.3	1310	**5**	**0.01***
	D	469	583.4	593.8	661	530.6	431.8	461.4	944		
Salpida	N	333	/	130.8	128.3	56.3	113.3	80.4	256	3	0.14
	D	195.6	347	331	234.2	86.4	260.9	323.6	240		
Pyrosomatidae	N	346.2	/	NA	NA	74.1	53.8	50	36	3	0.63
	D	50	NA	NA	NA	NA	115.7	NA	54		
Doliolida above 300 m	N	NA	/	36.8	27.3	35.1	61.7	32	35	2	0.14
	D	50	100	NA	110.6	200	78.7	50	17		
Doliolida below 300 m	N	646	/	625.6	633.9	600	450	600.1	56	2	0.19
	D	688.4	NA	695.5	596.1	621.1	500	600	37		
Actinopteri	N	303.3	/	346.2	386.5	331.1	238.2	365.5	1803	**1M**	**<0.01***
	D	568.1	489.9	529.8	521.9	510.7	466.4	497.1	1862		
